# Spatial and Temporal Characteristics of Insulator Contaminations Revealed by Daily Observations of Equivalent Salt Deposit Density

**DOI:** 10.3390/s150203023

**Published:** 2015-01-29

**Authors:** Ling Ruan, Ge Han, Zhongmin Zhu, Miao Zhang, Wei Gong

**Affiliations:** 1 State Grid Key Laboratory of High-Voltage Field-Test Technique, Electric Power Research Institute of Hubei Power Grid Corporation, Wuhan 430077, China; E-Mail: hbdky@sina.cn; 2 State Key Laboratory of Information Engineering in Surveying, Mapping and Remote Sensing, Wuhan University, Luoyu Road 129, Wuhan 430079, China; E-Mails: zhongmin.zhu@gmail.com (M.Z.); weigong@whu.edu.cn(W.G.); 3 College of Information Science and Engineering, Huazhong University of Science and Technology Wuchang Branch, Wuhan 430064, China; E-Mail: zm_liesmars@whu.edu.cn

**Keywords:** ESDD, on-line measurement, time series analysis, insulator contamination, power industry

## Abstract

The accurate estimation of deposits adhering on insulators is of great significance to prevent pollution flashovers which cause huge costs worldwide. Researchers have developed sensors using different technologies to monitor insulator contamination on a fine time scale. However, there is lack of analysis of these data to reveal spatial and temporal characteristics of insulator contamination, and as a result the scheduling of periodical maintenance of power facilities is highly dependent on personal experience. Owing to the deployment of novel sensors, daily Equivalent Salt Deposit Density (ESDD) observations of over two years were collected and analyzed for the first time. Results from 16 sites distributed in four regions of Hubei demonstrated that spatial heterogeneity can be seen at both the fine and coarse geographical scales, suggesting that current polluted area maps are necessary but are not sufficient conditions to guide the maintenance of power facilities. Both the local emission and the regional air pollution condition exert evident influences on deposit accumulation. A relationship between ESDD and PM_10_ was revealed by using regression analysis, proving that air pollution exerts influence on pollution accumulations on insulators. Moreover, the seasonality of ESDD was discovered for the first time by means of time series analysis, which could help engineers select appropriate times to clean the contamination. Besides, the trend component shows that the ESDD increases in a negative exponential fashion with the accumulation date (ESDD = a − b × exp(−time)) at a long time scale in real environments.

## 1. Introduction

Flashovers happening in high-voltage electric power transmission systems caused huge losses to society, estimated at 80–100 billion dollars just in the USA [1]. Contaminated insulators are considered a critical factor responsible for flashovers [2]. According to statistics from the power industry in China, contamination flashovers ranked second in the occurrence of power accidents and ranked first in the cost of power accidents in 2001 [3]. The increasing deterioration of air quality in China has drawn great attention from both the public and academia [4–6] and may also exert negative influences on insulator contamination accumulation [7]. Meanwhile, along with the booming economy both the scale and the operating voltage of the power grid has increased significantly in China. Hence, the damage caused by contamination flashovers would definitely be more serious unless appropriate measures are taken. To tackle this problem, periodic maintenance of insulators is indispensable for power transmission systems. However, such tasks are usually based on manual measurements of insulator contamination which are quite expensive and time consuming. Therefore, there is an urgent need to study the spatial and temporal characteristics of insulator contaminations.

The Equivalent Salt Deposit Density (ESDD) is accepted worldwide as a numeric index to describe insulator contamination. A negative relationship between ESDD and the leakage impedance has been proved and has been widely accepted as a basic rule to guide protection of the power grid [8–11], which means that the prediction of contamination flashovers requires precise determination of ESDD. In the last decade many researchers were dedicated to gaining insight into the characteristics of insulator contamination by means of ESDD observations. Salam proposed a model to estimate the influences of wind velocity on the ESDD [12]. Jiang carried out ESDD measurement experiments under different artificial pollution conditions to study the performances of various types of insulators [13]. Karamousantas tried to predict the ESDD by using artificial neural networks to schedule effective insulator maintenance [14]. Relative errors ranging from 5% to 16% were observed, which imply that the ESDD is predictable and calls for further endeavors to fill the gap between the current technology and the required accuracy. Su analyzed the mechanism of contaminant accumulation in heavily polluted coastal areas by measuring ESDD of samples collected from that area [15]. Liu indicated that the power transmission system is more vulnerable to contamination flashover at a given ESDD where there is ice adhesion on the insulator [16]. Sun studied the pollution accumulation on rail insulators in high-speed aerosols and found that the ESDD increased with the increasing wind velocity [17]. Wardman evaluated the influences of volcanic ash on flashover voltage of insulators with laboratory tests [18]. All the above studies focused on the contaminant accumulation on insulators in some specific scenarios, but there is still lack of basic understanding of the characteristics of the contaminants adhering on insulators in general and real environments. Till now, laboratory tests and measurements of samples collected from the real environment (*in-situ* measurements) are the major means to study the characteristics of the ESDD. However, laboratory tests are incapable of providing simulations of long-period real environments. Meanwhile, the *in-situ* measurements only offer monthly or even annual temporal resolution, so critical characteristics of the contaminant accumulation are very probably concealed in data with insufficient temporal resolution. In recent years, several groups have demonstrated novel sensors based on different technologies for automatically monitoring insulator contaminations on a daily basis, and such data have drawn extensive attention worldwide [11,19–22]. However, there is still a lack of analysis on these new data so that our understanding of the spatial and temporal characteristics of the ESDD cannot be improved.

To fill that gap, on-line daily measurements of the ESDD were collected from 16 sites in four different cities distributed across Hubei Province, in central China, to reveal the spatial and temporal characteristics of the ESDD in real environments. In Section 2, the background of the study area is presented. Along with that, the principle of the on-line measurements is also described. In Section 3, we report the original daily ESDD measurements and discuss their differences by taking the pollution conditions into considerations. In Section 4, the local polynomial regression fitting (LOESS) technique is introduced to apply time series analysis to the processed data. The long-term trends and seasonality of the ESDD are thus demonstrated. Owing to its low precision, we prefer to summarize by providing some qualitative rules rather than quantitative equations in the following sections.

## 2. Experimental Section

### 2.1. Study Area

**Figure 1.**
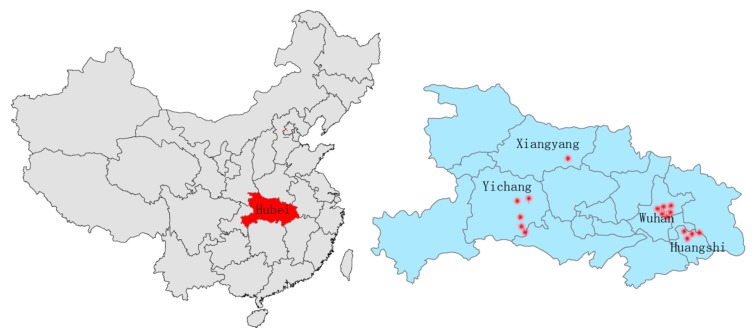
The location of Hubei in China and locations of monitoring sites in Hubei.

Hubei Province (Figure 1), with an area of 185,900 km^2^, is geographically situated between 29°05′–32°20′ N and 108°21′–116°07′ E in central China. The boundaries of the first and the second ladders of China cut through Hubei from north to south, which makes the topography vary significantly from east to west in Hubei. The provincial capital, Wuhan, is the political, economic, financial, cultural, educational and transportation hub of central China. The famous Three Gorges Dam and the Gezhou Dam, which are the origin of the West-East power transmission project, are both located in Yichang, in western Hubei. Hence, the robustness of the power grid is critical not only to the central China region but also to eastern China.

The on-line measurement sites for ESDD monitoring are distributed in four different districts, namely Wuhan, Yichang, Huangshi and Xiangyang which are abbreviated as W, Y, H and X, respectively (Figure 1). As the geographical coordinates of transmission line towers are classified according to Chinese laws, the names of relevant towers are labeled using the district name plus a serial number such as W1, Y1 and so on. Besides, we have added some random errors into the geographical coordinates so that the labels in relevant figures of this paper are roughly rather than exactly consistent with the geographical coordinates of the real towers.

### 2.2. The Principle of On-Line ESDD Measurement

The on-line ESDD measurements used in this study are based on optical technology. The quartz fiber rod is a kind of multi-mode dielectric waveguide, which is installed close to an insulator as a sensor. The basement membrane and high frequency mode of the quartz fiber transfers the optical energy. When there are no contaminants adhering on the quartz fiber rod, most (99%) of the energy is transferred in the core of the dielectric waveguide because the refractive index of the quartz is higher than that of the air. However, parts of the incident light would leak out if there is contamination, which has a higher refractive index, adhering on the surface of the rod, resulting in a decreased detected energy. Hence, the ESDD can be determined when decreases of the detected laser energy, the ambient temperature (T) and the relative humidity (RH) are measured using some inversion algorithms.

Firstly, there is a steady ratio of the ESDD on quartz rods and that on insulators according to many tests, which is the fundamental basis of on-line ESDD measurements using optical technology. Secondly, a nearly linear correlation between the ESDD and the loss of luminous flux (abbreviated as ϕ) has been witnessed at a fixed ratio of soluble deposit and non-soluble deposit (X) and a fixed RH. Thirdly, a evident relationship among ϕ, X and RH has also been confirmed. At last, a model is required to retrieve the ESDD. The model can be trained through samples collected by experiments in which ESDD is measured manually by traditional method. ϕ and RH are measured directly. X is estimated by using the variables ϕ, RH and T, then, the ESDD is retrieved.

**Figure 2.**
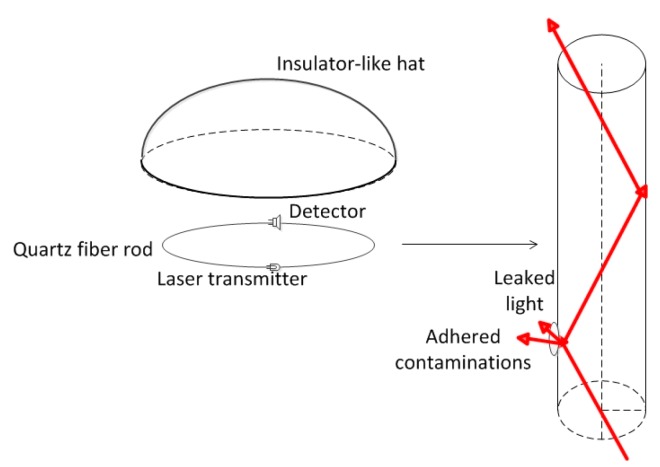
Sketch of the on-line measurement apparatus.

The apparatus (ODU-11, Kangpu, Wuhan, China, Figure 2) involved in this study is designed to measure the ESDD for power transmission lines varying from 110 kV to 1000 kV. Its operating ambient temperature and relative humidity vary from 233.15 to 358.15 K and 0% to 100%, respectively. Its measurement range is 0–1 mg/cm^2^ with an accuracy of 10%.

## 3. Results and Discussion

### 3.1. Spatial Heterogeneity of the ESDD

**Figure 3.**
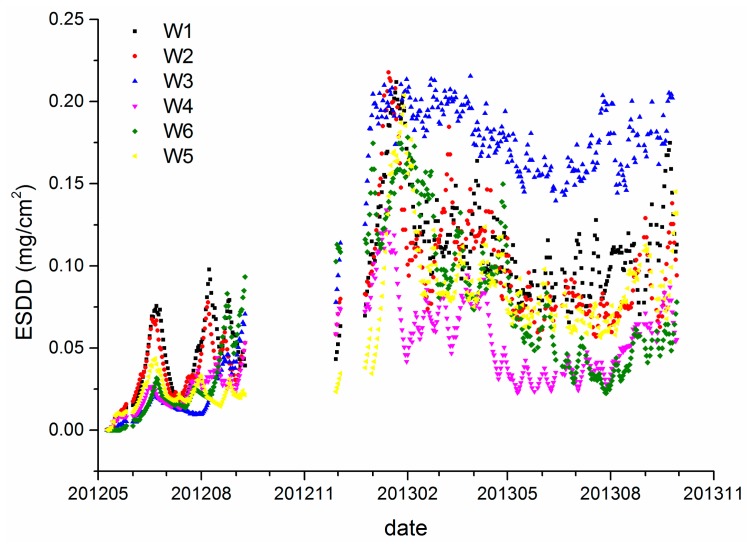
On-line measurements of six sites in Wuhan.

Figure 3 shows the daily ESDD acquired by the on-line measurement apparatus from six sites in Wuhan. The monitoring project started in 2011. There are very sparse measurements before April 2012. Hence, the data shown in Figure 3 start from that time. Besides, there are two gaps in the observation sequences because of apparatus maintenance. The averages of W1 to W6 are 0.09, 0.08, 0.13, 0.05, 0.07 and 0.07 mg/cm^2^, respectively, with standard deviations of 0.046, 0.045, 0.028, 0.048, 0.042 mg/cm^2^, respectively.

**Figure 4.**
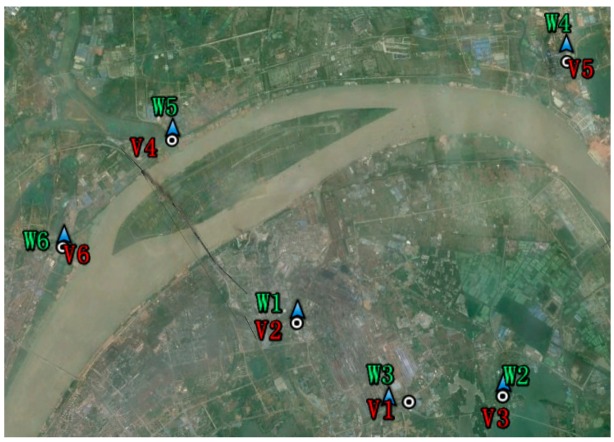
Distributions of six sites in an image of Wuhan acquired by satellite. The blue triangle with green labels represents the ESDD monitoring site, while the white annulus with red labels represents the verification point. The dimensions of the area shown in Figure 4 are roughly 10 km by 10 km.

The size of the area shown in Figure 4 is about 10 km by 10 km. Hence, any influences of meteorological factors can be regard as homogeneous. Differences of the mean ESDD are probably attributable to different pollution sources near each site. W1, W2 and W3 are close to the Wuhan Iron and Steel Corporation (WISCO) which is one of top three steel companies in China. Figure 3 also shows that the ESDD of W3 is evidently larger than the others. Our field investigation verified that this site is in the vicinity of a major WISCO steel mill and an expressway. The results imply that the contamination accumulation on insulators is very susceptible to local emissions at a fine spatial scale. Therefore, the current polluted area map of which the spatial resolution has not been strictly defined and is often coarser than 50 km is insufficient to reflect the real conditions of insulator contamination.

Besides, though either the mean ESDD or visual interpretation to Figure 3 is capable of distinguishing the contaminant accumulation level of each site, such differences are very likely concealed by traditional ESDD measurements which are based on annual even biennial power grid maintenance. Figure 3 demonstrates that the rank ordering of ESDD measured at different sites varied with the monitoring data significantly. This is likely due to different local air pollution and meteorological conditions. Consequently, the assessment of pollution level which is based on annual or biennial measurements depends largely on the sample date. From the perspective of estimation of spatial distribution of ESDD, the on-line measurements have an evident advantage over the traditional means.

**Figure 5.**
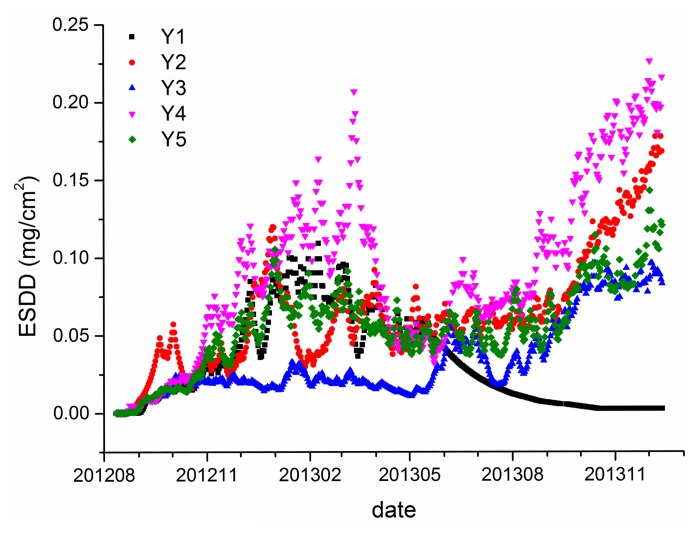
On-line measurements of five sites in Yichang.

**Figure 6.**
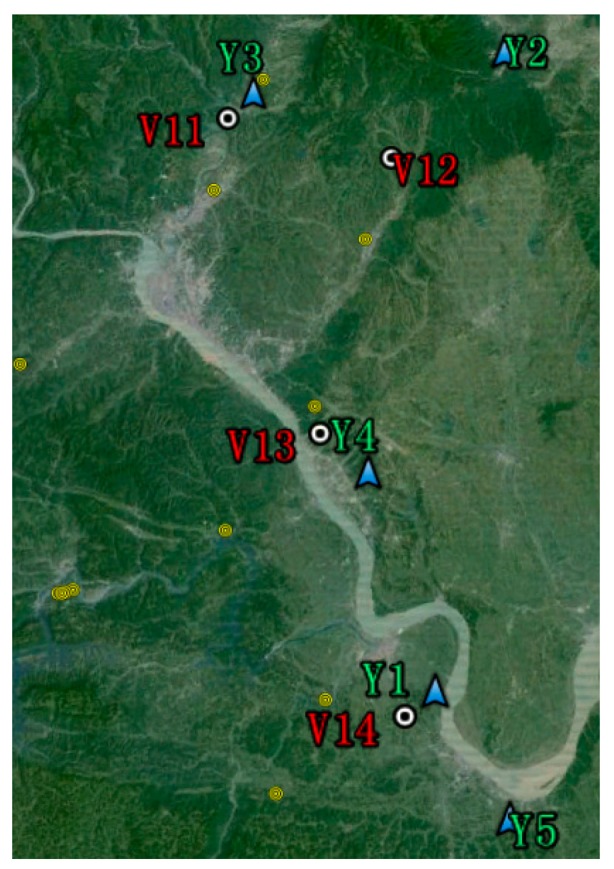
Distributions of six sites in an image of Yichang acquired by satellite. The legend is consistent with that of Figure 4. The yellow circles are other verification points which are far away from monitoring sites. The dimension of the area shown in Figure 6 is roughly 50 km by 80 km.

To verify the above discoveries, the on-line ESDD measurements and sites' distributions in Yichang are shown in Figures 5 and 6. The monitoring time span is from August 2012 to December 2013. Though the monitoring period of Yichang district is shorter than that of Wuhan, the integrality of the data set of Yichang is superior to that of Wuhan. There is an obvious fault in Y1 after May 2013 because the smooth decline of ESDD is inaccurate according to Figure 5. We have reported the abnormal occurrence to the relevant maintenance department and they have confirmed that the sensor of Y1 is out of order. Hence, such an incident proves that the on-line ESDD measurements have the potential to carry out quality assessments by themselves rather than by sampling logs which are often inaccurate in practice.

The averages of Y2 to Y5 are 0.06, 0.03, 0.09 and 0.06 mg/cm^2^, respectively, with standard deviations of 0.037, 0.026, 0.054 and 0.027 mg/cm^2^, respectively. Again, an evident spatial heterogeneity is witnessed as shown in Figures 5 and 6. Two reasons may be responsible for the fact that the ESDD distribution in Yichang is more heterogeneous than that in Wuhan. Firstly, the four valid sites in Yichang are distributed in a larger area of 30 km by 80 km. Secondly, Wuhan is in the Jianghan Plain whereas Yichang is close to the Three Gorges area where mountain land is the dominant topography. Moreover, we have carried out a field investigation in Yichang and found that the pollution levels of these sites are consistent with their mean ESDD. Y4 is located in an industrial district where the quarrying industry is very developed, whereas, Y3 is located in a county where wine-making is the main industry. Y2 and Y5 share similar conditions. There are heavy industries which are about 15 km away from either Y2 or Y5.

**Figure 7.**
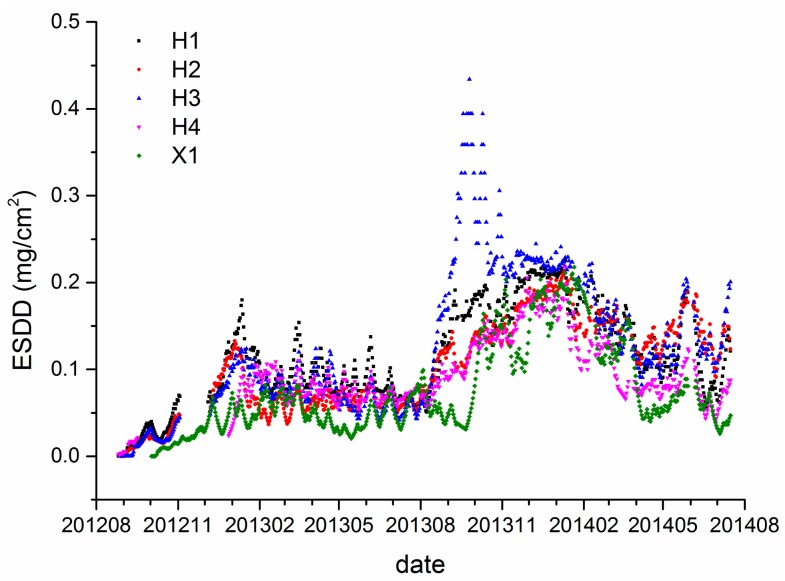
On-line measurements of five sites in Huangshi and Xiangyang.

**Figure 8.**
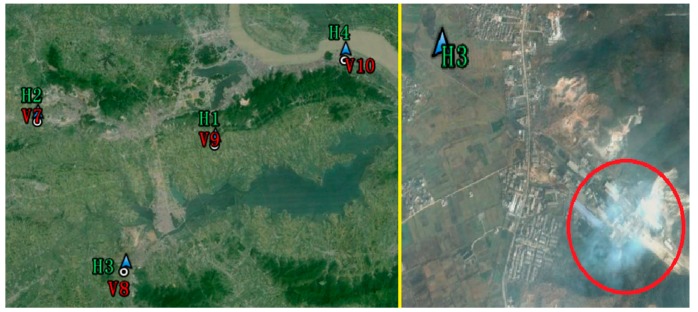
Distributions of four sites in an image of Huangshi and high resolution imagery acquired by satellite in the vicinity of H3. The dimensions of the left and right parts is about 25 km by 35 km and 1.5 km by 1.5 km, respectively.

Figure 7 shows the on-line ESDD measurements from Huangshi and Xiangyang. It would be lengthy to further analyze the relationship between the ESDD and pollution levels. However, a singular phenomenon is immediately noticed in the on-line measurements of H3. There is an abnormal fluctuation in the curve of H3 between late September and early November in 2013. According to satellite imagery, H3 is the only site among all 16 sites which is located in a large area of farmland. To our knowledge, peasants in Hubei have habit of burning stalks in autumn to increase the fertilization of the fields though previous research has shown no significant effects. It is speculated that the abnormal fluctuation probably are attributable to the biomass burning near the H3 site. Figure 8 shows the land cover near H3. Coincidentally, a plume of smoke was captured by the passing by satellite. There is no direct evidence to blame biomass burning for the dramatic rise of ESDD because the acquired date of the imagery shown in Figure 8 is not consistent with the date of the incident. However, Figure 7 proves that there are indeed some biomass burning incidents near H3. Consequently, we strongly suspect that the singular phenomenon in Figure 8 is related to the biomass burning. Fontana has proved that the biomass burning during harvest periods indeed exerts an evident influence on insulator contamination using another ESDD on-line measurement technique in Brazil [21]. Hence, it is recommended that maintainers of power transmission lines should pay great attention to insulators that are located in farmlands in autumn.

The above analysis has shown that the spatial heterogeneity can be seen at a spatial scale of several kilometers, a fine scale in geography. As the distances between Wuhan, Yichang and Huangshi are 80–340 km, it is possible to study the spatial heterogeneity at a regional scale. The Kruskal-Wallis test is utilized to verify whether the regional scale is appropriate for assessments of the spatial heterogeneity of ESDD. Because there is only one site in Xiangyang, it is thus inadequate to apply the Kruskal-Wallis test for that region. The other three regions are involved in this test. The hypothesis is that ESDD varies significantly at a regional scale. The resultant *p*-value is 0.03621, smaller than 0.05. In other words, we have 96.38% confidence to confirm that the spatial heterogeneity of ESDD can be also observed at a regional scale. This also explains why polluted area maps of which the spatial scale is always tens or even hundreds of kilometers are widely accepted and used by the power industry in China.

### 3.2. Relationship between ESDD and Air Pollution

On-line measurements provided by 16 sites in Hubei prove the spatial heterogeneity of ESDD at both the fine and coarse spatial scales and that it is very likely related with the nearby pollution levels. To verify the relationship between the ESDD and air pollution, a specific field investigation was carried out in Wuhan and Huangshi. Regarding the Yichang area, we have also collected data from another field investigation where the aim was not to investigate the on-line ESDD monitoring sites. For this reason, the verification points in Figure 8 are not very close to the monitoring sites. In these field investigations, mass concentrations of ambient PM_10_ (particulate matter with an aerodynamic diameter smaller than or equal to 10 μm, a widely accepted air pollution index) were recorded along with temperature, pressure, relative humidity and wind velocity. Results are shown in Table 1.

**Figure 9.**
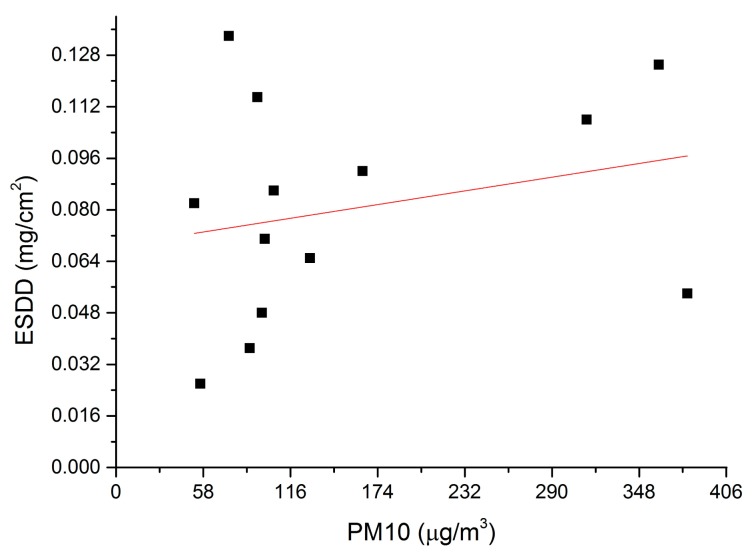
Correlation between the mean ESDD and instantaneous PM_10_, where the red line is the linear regression equation.

According to Table 1 and Figure 9, there is a positive correlation between the ESDD and PM_10_. However, the *R*^2^ of the regression equation is very low, implying that the equation should not be utilized to estimate ESDD by using PM_10_. Three reasons are responsible for this result. Firstly, the mean ESDD is influenced by long period mean PM_10_. It is still doubtful that the instantaneous PM_10_ is suitable to represent the annual PM_10_ though we have cautiously selected the date of the field investigation to avoid evident disturbances. Secondly, the air pollution composition varies geographically and the responses of the insulator contamination to different chemical substances are different. For instances, W4 and W5 share similar PM_10_ concentrations while their major pollution sources are quite different. An evident difference on ESDD between W4 and W5 is seen, implying that the insulator contamination may be very susceptible to pollution emitted by power plants. Thirdly, the particulate matter with larger aerodynamic diameter is not taken into consideration. PM_10_ represents particulate matter that is able to enter human lungs, which is thus harmful for health. However, for the insulator contamination, those particulate matters with larger aerodynamic diameter are equally important. It is noticed during the field investigation of V3 that the filter membrane is as black as that observed at V2, though the measured PM_10_ at V3 is much lower. this means we cannot simply conclude that the pollution level of V3 is lower than that of V2 by just comparing the PM_10_ mass concentrations of two points.

Furthermore, we have collected long-term PM_10_ observations from Hubei Environmental Monitoring Central Station (HEMCS, Wuhan, China) to match the daily ESDD measurements in Wuhan. The distribution of environment monitoring stations in Wuhan can be found in reference [5]. Only data from the Wujiashan station (Wuhan, China) which is about 23 to 40 km away from ESDD sites were available to us. Because the environmental monitoring stations don't coincide with ESDD sites geographically, we have to build a relationship between averages of six ESDD sites and air pollution data. As mentioned above, the daily ESDD measurement contains an error of 10%. Moreover, the lag effect of air pollution on insulator contamination is still unknown. Consequently, we utilize monthly data to build the relationship between ESDD and PM_10_.

**Figure 10.**
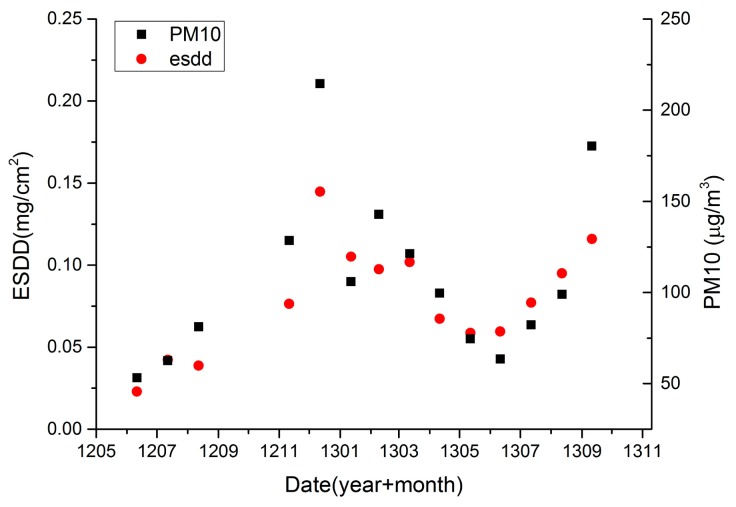
Monthly-averaged ESDD and PM_10_ observations.

Figure 10 shows the processed ESDD and PM_10_ data. A very evident positive relationship between monthly-averaged ESDD and PM_10_ is shown in Figure 10. A linear regression equation with *R*^2^ of 0.78 can be built. Therefore, it is concluded that the relationship between ESDD and PM_10_ is proved at a regional scale and a monthly scale. Lasting monitoring of air pollution at each ESDD on-line measurement site would be very helpful to reveal numeric relationships between ESDD and PM_10_ at a local scale and a daily scale.

### 3.3. Temporal Variability of ESDD

Figures 3, 5 and 7 demonstrate that spatial heterogeneity can be easily concealed by monthly- or annual-interval measurements. The evident temporal variability which has already been shown in Figure 10 is responsible for that phenomenon. The standard deviation of each site is rarely lower than half of the corresponding average. In other words, the ESDD fluctuates so wildly that the spatial heterogeneity tends to be ignored when comparing with the temporal variability. Meanwhile, the current periodical maintenance of power transmission line pays little attention to selection of appropriate operating times by taking periodical variations of deposit accumulation into consideration. We have also collected several ESDD measurements that are acquired when periodical maintenance was carried out by means of the traditional method. It is noticed that the sample time covers nearly every month in a year, suggesting that the current periodical maintenances are not scheduled according to the temporal characteristics of the deposits adhering on insulators. Consequently, it is extremely critical to study its temporal characteristics to guide the rational selection of maintenance dates in the future.

First of all, we assume that the temporal variability of ESDD is related with climatic factors which are homogenous at a large scale. Though the above hypothesis has not been witnessed by any previous study, precipitations and wind can polish insulators in principle. Besides, the temporal variability of air pollution has already been verified by many researchers, and it is dominated by climatic factors [23–25]. Moreover, the ESDD depends highly on the regional air pollution level according to previous discussions and studies [17,18,26]. Therefore, we think that the hypothesis is reasonable. The mean daily ESDD in a region is calculated by averaging the daily measurements of several sites in a district. Besides, geographical averaging would also makes the temporal variability steadier, being free of local periodical influences.

**Figure 11.**
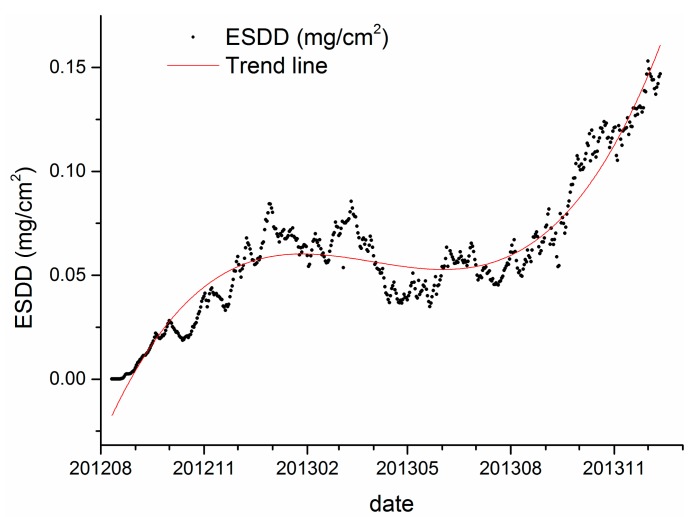
Mean daily ESDD and its trend line in Yichang.

Furthermore, Figures 3 and 5 illustrate that there are many burrs in the curve of daily ESDD measurement. However, we have inadequate confidence to distinguish which parts are meaningful signals and which parts are merely noises. In our opinion, the absolute accuracy is the major drawback of on-line measurements. Only time series of measurements is meaningful and robust for scientific research, so would be risky to draw any conclusion from an individual measurement. The data of Wuhan thus has to be abandoned because of lack of integrality regarding the monitoring span. Figure 11 shows the average daily ESDD of Yichang and the corresponding trend line calculated by a three order polynomial function.

The adjusted R square of the fitting equation is 0.91, indicating that 91% of the ESDD temporal variability can be explained by the trend line. Figure 11 illustrates that there is an increasing trend and a seasonal variation in the ESDD curve. In general, the ESDD rises with the increasing number of observation days. Meanwhile, the growth of ESDD is variable rather than constant with the accumulation days. The ESDD grows faster in autumn than in other seasons. The ESDD even decreases slightly in summer. Figure 11 also illustrates that the oscillation period of ESDD is 12 months.

A conclusion that can be drawn from Figure 11 is that the plot of ESDD is a combination of an increasing trend component and a seasonal variation component. Hence, the Seasonal and Trend decomposition using Loess (STL) is introduced to distinguish the two components from each other. The STL technique is widely utilized by environmental scientists [27,28] and economists [29,30] to deal with scientific and economic issues. STL is a filtering procedure for decomposing a time series into trend, seasonal and remainder components. Its advantages includes specification of amounts of seasonal and trend smoothing that range, in a nearly continuous way, from a very small amount of smoothing to a very large amount; robust estimates of trend and seasonal components that are distorted by aberrant behavior in the data; and the ability to decompose time series with missing values [31]. All statistical data analyses have been performed by the software environment R 3.1.1(R Development Core Team, Vienna, Austria, 2014).

It is worth noting that two periods are the minimum requirement to carry out the STL. Hence, the duration of on-line measurements should be longer than two years to satisfy the technical demands. Data of both Wuhan and Yichang cannot fulfill this demand, however, the monitoring periods of the Huangshi and Xiangyang sites are longer than 2 years, spanning from September and October 2012 respectively to October 2014. Though the monitoring data is not absolutely continuous at a daily scale as shown in Figure 7, continuous monthly data, which is calculated from the daily data, is sufficient and appropriate to apply the decomposition. Actually, the STL technique is capable of dealing with time series with missing values as mentioned previously. It is worth mentioning that monthly data is used to retrieve the trend and seasonality components because such a temporal scale is more appropriate than a daily scale to achieve our goal. Moreover, it is not certain that the time series of ESDD that is measured once a month is adequate to apply the STL. The monthly data which is based on averages of daily measurements is completely different from that measured once a month. Figures 3, 5, and 7 demonstrate that there are many fluctuations on the daily ESDD curves. Provided that we measured the ESDD right at the peak a fluctuation in one month and measured the ESDD at the foot of another fluctuation in the following month, the seasonality would then be concealed by such samples.

**Figure 12.**
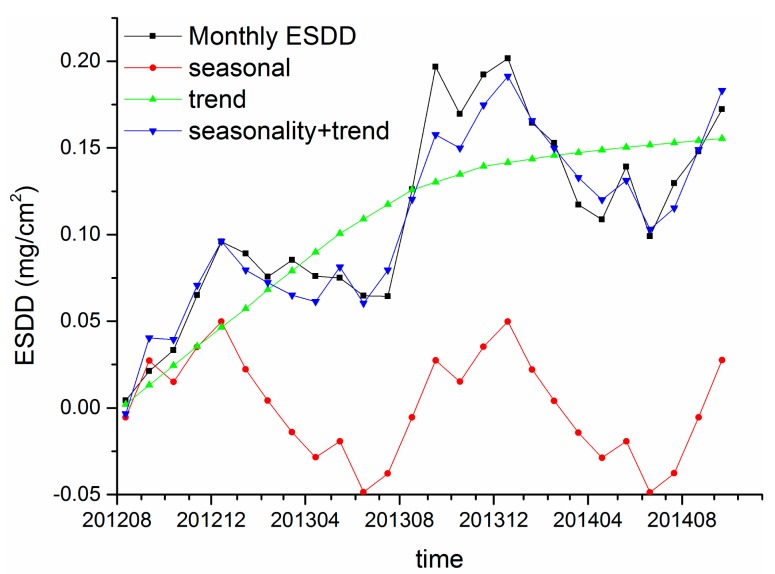
Decomposition results of Huangshi sites.

**Figure 13.**
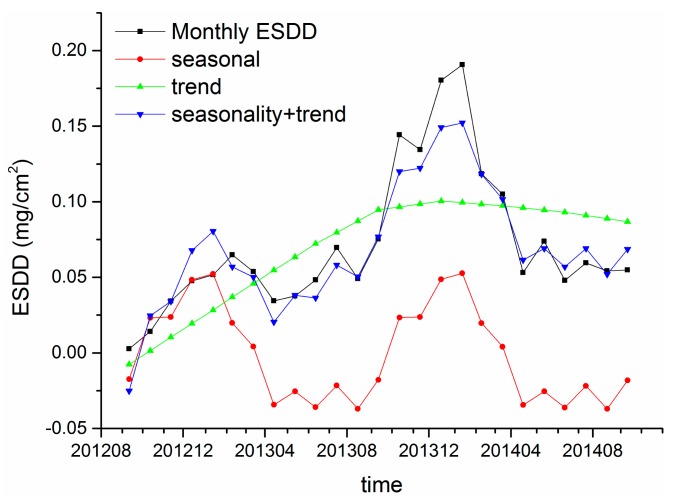
Decomposition results of Xiangyang site.

Figures 12 and 13 demonstrate the resultant components of Huangshi and Xiangyang sites, respectively. The blue lines and black lines are very similar, indicating that the reminder is acceptable. Therefore, the results of time series analysis are accurate and reliable. Regarding the ESDD trend, an evident relationship between the ESDD and accumulation time is revealed at a large time scale, which can be expressed as ESDD = a − b × exp(−t). Many tests in artificial environments show such relationships at a time scale of hours or days. To our knowledge, the ESDD trend at the time scale of years has not been reported yet by using numeric analysis.

Regarding the seasonality of ESDD, a periodical oscillation is witnessed in Figures 12 and 13. The ESDD rises in autumn and declines in spring reaching its peak in winter and falling to a minimum in summer. Two reasons may be responsible for the seasonality of ESDD. In Hubei, a typical subtropical monsoon climate results in abundant precipitation from May to July [32]. The insulator contamination would decrease in this period. However, it is beneficial to contaminant accumulations from September to January when heavy rain seldom happens. The other reason is the seasonality of near surface air pollution. The long-term observations from HEMCS indicate that hazes occur frequently in winter and late autumn in Hubei. Song also witnessed such a seasonality of PM_10_ in China by using satellite products [33]. Figure 10 also illustrates that the time curves of PM_10_ and ESDD are similar. In conclusion, the heavy air pollution in autumn and winter would accelerate accumulation of insulator contamination.

Though Huangshi is over 300 km away from Xiangyang, a similar trend and seasonality are witnessed for the accumulation of insulator contaminations in the two regions. Therefore, the above trends and seasonality may be universal rules for the accumulation of insulator contaminants in inland China, especially in regions that share similar climatic characteristics. Moreover, Figures 12 and 13 illustrate that the seasonal curves are different in the two regions, implying that there are some factors influencing the temporal characteristics of insulator contamination. It is speculated that meteorological factors, such as the precipitation, the wind velocity and *etc.*, may be the dominant factors which determinate the seasonality of ESDD, while environmental factors, such as PM_10_, gaseous pollutants and *etc.*, may be the dominant factors which determinate the ESDD trend.

Finally, it is worth noting that the duration periods of both the Huangshi and Xiangyang sites are not long enough to draw any quantitative conclusions. Long-term observations of daily ESDD will be definitely critical for a further and more detailed study. Besides, observations from more regions are necessary to verify the discovered rules and to further study characteristics of deposits adhering on insulators.

## 4. Conclusions

In this study, we collected daily ESDD from 16 monitoring sites located in Hubei Province, China, acquired by on-line measurement apparatus which utilize optical technology. There are many fluctuations on the daily ESDD curves containing both signals and noises owing to the low precision of the relevant apparatus. However, the time series of ESDD observations is proved to be able to reflect both the spatial heterogeneity and temporal variability of insulator contamination. It is seen that the ESDD largely depends on the surrounding environment. The spatial heterogeneity of ESDD is verified at regional scale by using the Kruskal-Wallis test. Meanwhile, in an area of 10 km by 10 km, evident differences of ESDD observations are also found, indicating that current polluted area maps which are widely adopted as key means to guide the periodic maintenance of power facilities in China should be improved on a spatial scale to reflect the real distribution of insulator contamination, especially in urban areas. Furthermore, the assessment on levels of insulator contamination is meaningless without consideration of its temporal variability because the amplitudes of the temporal variability are large enough to conceal any spatial heterogeneity. Moreover, the ESDD time series is decomposed into the trend component and the seasonality component by using LOESS. It is first verified in real environments that there is a negative exponential function between the ESDD and the accumulation time at the time scale of years. Besides, an evident seasonality of ESDD is discovered by means of monthly averaged ESDD and LOESS. To our knowledge, such a rule has not been reported previously and it will play an important role in scheduling periodic maintenance of power facilities. Finally, on-line daily ESDD measurements of a longer are urgently needed, and more locations and higher accuracy data should be collected and analyzed to reveal more robust and accurate rules quantitatively.

## Figures and Tables

**Table 1.** Field investigation results.

**Monitoring Site**	**Verification Point**	**ESDD ^a^ (mg/cm^2^)**	**PM_10_ (μg/m^3^)**	**Land Use**	**Major Pollution Source ^b^**
W1	V2	0.092	164	factory	iron mill
W2	V3	0.082	52	road	vehicle exhaust
W3	V1	0.125	361	road	iron mill
W4	V5	0.048	97	bare land	vehicle exhaust
W5	V4	0.071	99	factory	power plant
W6	V6	0.065	129	bare land	vehicle exhaust
H1	V9	0.115	94	road	vehicle exhaust
H2	V7	0.108	313	road	cement plant
H3	V8	0.134	75	farmland	mine field
H4	V10	0.086	105	road	power plant
Y1	V14	error	231	orchard	cement plant
Y2	V12	0.064	89	orchard	cement plant
Y3	V11	0.034	56	orchard	distillery
Y4	V13	0.090	380	orchard	chemical plant
Y5	no data	0.056	no data	no data	no data
X1	no data	0.073	no data	no data	no data

a The ESDD hereby is the average of all daily observations during the monitoring period;

b There is not a strict definition on sphere of influence. We search around each verification point to identify pollution sources. Some are 10 m away from the verification point while others are several hundred meters away. The max search radius is 1 km.
